# An exploratory study of factors that affect the performance and usage of rapid diagnostic tests for malaria in the Limpopo Province, South Africa

**DOI:** 10.1186/1475-2875-6-74

**Published:** 2007-06-02

**Authors:** Devanand Moonasar, Ameena Ebrahim Goga, John Frean, Philip Kruger, Daniel Chandramohan

**Affiliations:** 1 Disease Control and Vector Biology Unit, London School of Hygiene and Tropical Medicine, London, UK; 2 Southern African Fogarty AIDS International Training and Research Programme (AITRP), The Centre for the AIDS Programme of Research in South Africa (CAPRISA), Durban, South Africa; 3 Parasitology Reference Unit, National Health Laboratory Service, Johannesburg, South Africa; 4 Malaria Control Programme, Limpopo Department of Health and Social Development, Tzaneen, South Africa

## Abstract

**Background:**

Malaria rapid diagnostic tests (RDTs) are relatively simple to perform and provide results quickly for making treatment decisions. However, the accuracy and application of RDT results depends on several factors such as quality of the RDT, storage, transport and end user performance. A cross sectional survey to explore factors that affect the performance and use of RDTs was conducted in the primary care facilities in South Africa.

**Methods:**

This study was conducted in three malaria risk sub-districts of the Limpopo Province, in South Africa. Twenty nurses were randomly selected from 17 primary health care facilities, three nurses from hospitals serving the study area and 10 other key informants, representing the managers of the malaria control programmes, routine and research laboratories, were interviewed, using semi-structured questionnaires.

**Results:**

There was a high degree of efficiency in ordering and distribution of RDTs, however only 13/20 (65%) of the health facilities had appropriate air-conditioning and monitoring of room temperatures. Sixty percent (12/20) of the nurses did not receive any external training on conducting and interpreting RDT. Fifty percent of nurses (10/20) reported RDT stock-outs. Only 3/20 nurses mentioned that they periodically checked quality of RDT. Fifteen percent of nurses reported giving antimalarial drugs even if the RDT was negative.

**Conclusion:**

Storage, quality assurance, end user training and use of RDT results for clinical decision making in primary care facilities in South Africa need to be improved. Further studies of the factors influencing the quality control of RDTs, their performance of RDTs and the ways to improve their use of RDTs are needed.

## Background

The South African National Malaria treatment guidelines stipulate that malaria treatment (using artemesinin-based combination therapy) should be based on definitive diagnosis using microscopy or malaria rapid diagnostic tests (RDTs) [1]. South Africa has been implementing RDTs to diagnose malaria within malaria endemic areas since 2001[2]. In a primary health care setting, RDTs are most appropriate: they are easy to use, do not require sophisticated technology and give rapid results [3]. The functioning and accuracy of RDTs can be affected by several factors, including manufacturing defects, storage, transport, and end-user performance [4]. Malaria diagnostic tests need to be highly accurate because false negative and false positive diagnoses have medical, social, and economic consequences such as prolongation of illness, increase in morbidity and mortality and loss in credibility of health services [5,6].

**Figure 1:**
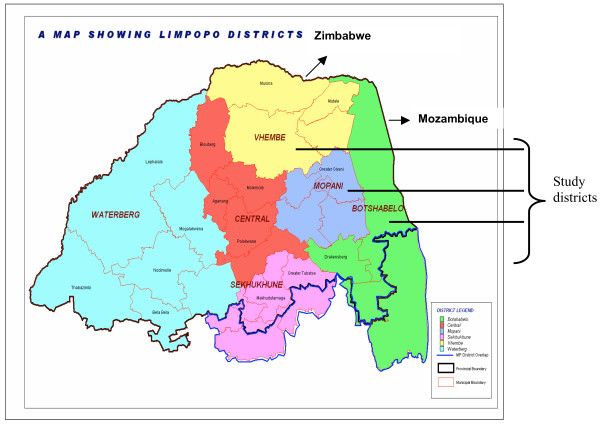
Magisterial Districts of the Limpopo Province.

The Limpopo Province, is one of three malaria endemic provinces in South Africa and has the highest malaria incidence [2]. Figure 1 provides a map showing magisterial areas. Although RDTs was introduced for malaria diagnosis in Limpopo in 2003, operational issues relating to its performance and use have not been rigorously investigated. A study was therefore undertaken in the Limpopo Province to determine which factors affected quality and usage of RDTs.

**Figure 2:**
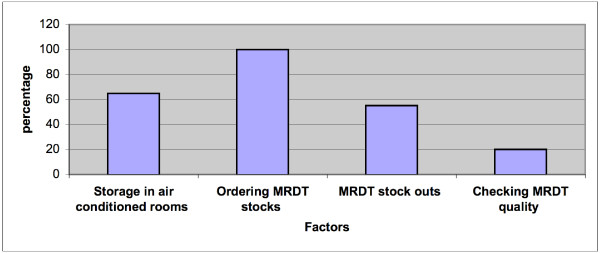
Graph Showing factors affecting the quality and usage of RDTs.

## Methods

Purposefully selected key informants and randomly selected nurses at the primary health facility level were interviewed using a semi-structured questionnaire. Key informants included three hospital pharmacy staff, one regional pharmacy manager, three district malaria managers, one provincial malaria control manager and two researchers. Among the nurses interviewed, 17 were from primary health care (PHC) clinics and three were from hospitals.

A two-stage sampling procedure was used to select PHC clinic staff [7]. All clinics and health centers within the three malaria-affected districts were listed. Clinics and health centers with fewer than 10 malaria cases per annum were excluded from the sampling frame. In the first stage of sampling, 10% of the clinics (10/100) and 20% of health centers (7/35) within each selected district were randomly selected. In the second stage, one nursing sister was randomly selected from each selected health facility. Interviews were transcribed, ordered and coded in matrixes, using the key categories of procurement & stock monitoring, storage, transport, quality control and end user experiences [8].

### Ethics

The University of Limpopo Research Ethics Committee, the Limpopo Department of Health and Social Development and the London School of Hygiene and Tropical Medicine granted ethical permission for this study. Informed consent was acquired from all interviewees.

## Findings

### Procurement and stock monitoring

In all health facilities, there was evidence that stocks and expiry dates were monitored regularly. Stock monitoring methods ranged from stock cards (paper based) to electronic systems. Nursing staff were aware of the seasonal increase in malaria cases and stated that they ordered more RDT stock before and during the season; however, only 20% of the nurses interviewed were able to accurately give the limits of the malaria season (September-May). More than half (55%) of the nurses indicated that stock-outs of RDTs occurred. However, they reported that this was rare (one or two times in a season) and contingency plans existed to replace stock from either the nearest clinic or hospital pharmacies. Replacement of stock took place within 24 hours. District malaria managers reported RDT stock-outs in clinics in the 2005/2006-malaria season. They responded by alerting the necessary authorities (district hospital or regional pharmacy depots) or transporting malaria kits to facilities.

Pharmacy staff reported deploying pharmacy assistants to some health facilities to assist with ordering of pharmaceuticals including RDTs. One hospital had a bar code system for ordering pharmaceutical supplies. At the regional pharmacy level an electronic system is in place to increase stock as demand increased, and in most cases this is proportional to the seasonal increase in malaria cases.

### Storage of RDTs

Sixty five percent (13/20) of the nurses reported that RDTs were stored correctly i.e. in an air-conditioned room with regular temperature monitoring. Among the nurses from the seven facilities that did not implement correct storage, three (42%) were very concerned with temperature fluctuation as thermometers were unavailable for temperature monitoring. The remaining 4/7 (53%) indicated that although their clinics lacked air conditioning, room temperature was monitored, and that it rarely rose above 30°C. This however was not corroborated with any recorded data.

Malaria managers (district and provincial) accepted that some clinics did not keep RDTs in a cool environment; however they were not concerned about this, commenting that "*the kits did not stay in the clinics for too long*", due to their frequent use.

Pharmacy managers in hospitals and regional depots stored RDTs in an air-conditioned environment (temperature range 15–25°C). Monitoring charts were produced when requested by the interviewer.

### Quality control of using RDTs

Only 4/20 nursing staff said that they checked the quality of RDTs, this they reported to have been done, by comparing the agreement between a diagnosis based on clinical signs and symptoms and the RDT result. Two of 20 nurses reported that they used blood smear results to confirm the RDT results occasionally. Three (15%) nurses reported that they gave antimalarial drugs to RDT negative patients if the clinical presentation was suggestive of malaria.

**Figure 3:**
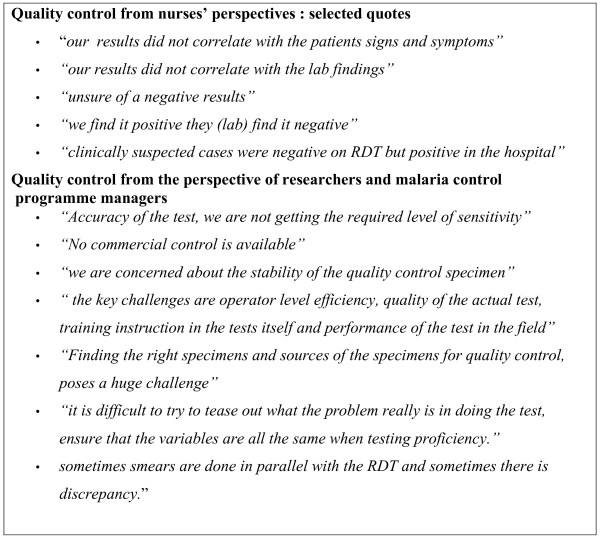
Quality-related challenges: selected quotes by nurses, researchers and malaria control managers.

Figure 3 highlights the key challenges identified by nurses, researchers and malaria managers relating to quality.

In summary, managers were very concerned that RDTs quality were not being monitored at health facility level. One informant stated, "*RDT quality control, both at the manufacturing side and at the testing stage, was lacking. The key is the end user's ability to distinguish between positive and negative results."*

### End User experiences in using RDTs

**Figure 4:**
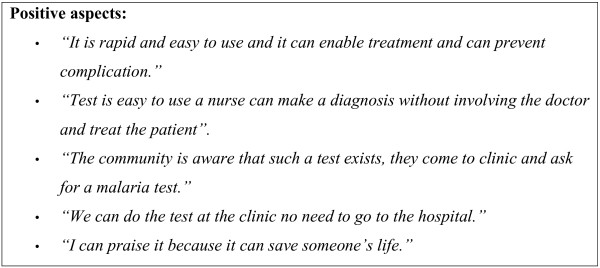
Nurses quotes on positive aspects of RDT and Summary of negative aspects of the RDTs.

Nursing staff had huge praise for the use of the RDTs, Figure 4 highlights nurses' responses on positive aspects of using the RDTs.

More than half the nurses (12/20) however reported that they did not receive external training on RDT use; however, 10 nurses reported receiving in-house training. Almost all (19/20) nurses said that they were confident in using the RDT.

The accuracy of RDT result was the key concern for 35% of the respondents, false positive and negatives were stated as major challenges.

### Managers and laboratory technologists' views

Malaria managers commented that RDT readings were problematic – "*the fault could have been due to the clinic staff not reading the test in time*." One laboratory technologist said that the nurses were not doing the test properly – "*When we receive the test back we see that they are putting too much blood*."

## Discussion

Although this study involved a small representative sample of users of RDT at the primary care facilities in South Africa, it has revealed that (1) there are sporadic problems of stock-out of RDT during peak transmission seasons; (2) the storage facilities and monitoring is inadequate in many primary care facilities; (3) the accuracy of the RDT was tested on an *adhoc *basis leading to nurses sometimes offering antimalarials to RDT-negative cases on the basis of their clinical judgment; (4) there is a possibility that RDTs under-diagnose malaria in the current context and (5) there is very limited training on the use of RDT at the PHC level.

According to the WHO, temperatures above 30°C are considered inappropriate for storing RDTs [4]. As temperatures above 30°C can affect overall performance of the RDTs, temperature-monitoring needs to take place in all clinics and environments. Air-conditioning or similar cooling equipment should be considered in those clinics that exceed the WHO recommended threshold [4]. RDT should be stored in a centralized store as long as possible and care should be taken during transport and storage at the health facilities to minimise degradation. Use of positive control wells and temperature monitors should be considered in South Africa to assure the quality of the RDTs and to build confidence of the users on RDT [9-11].

The quality of the RDT can be established at three levels: post-manufacture level, end user level and through the use of positive control wells [9,12]. Due to the uncertainty of the quality of the test and lack of confidence in some cases of interpreting the results, patients were getting inappropriate treatment. For example some cases were given antimalarial treatment on clinical diagnosis even if the RDT was negative. It is possible to obtain false negative RDT results [6]. However there is no system of evaluating the performance of RDT in the routine health services and to build confidence among the users of RDT. Very few users at the primary health care clinics were formally trained in performing and interpreting RDTs. Thus an in-service training and quality control system is needed urgently to ensure appropriate use of RDTs and effective treatment of malaria in South Africa. Although package inserts are useful it would be easier for the end-user to have posters or job aids so that the test procedure can be easily visible and read especially during busy periods and late in the night [13-15]. End user proficiency testing such as those described in other studies may be considered[13,14,16].

## Conclusion

This study in South Africa may not be applicable to other countries in Africa. However, the lessons on storage, quality assurance and training observed in this study would be applicable to most settings where RDTs are introduced. Further studies of the factors influencing the appropriate use of RDTs and the ways to improve the use of RDTs are needed in the study setting.

## Authors' contributions

All the authors conceptualized the study, participated in the analysis, drafting of the manuscript and writing the final version of the paper.
